# A review of the economic burden of ADHD

**DOI:** 10.1186/1478-7547-3-5

**Published:** 2005-06-09

**Authors:** Louis S Matza, Clark Paramore, Manishi Prasad

**Affiliations:** 1 The MEDTAP Institute at UBC, 7101 Wisconsin Ave, Suite 600, Bethesda, MD, 20814 USA

## Abstract

Attention-deficit hyperactivity disorder (ADHD) is a common disorder that is associated with broad functional impairment among both children and adults. The purpose of this paper is to review and summarize available literature on the economic costs of ADHD, as well as potential economic benefits of treating this condition. A literature search was performed using MEDLINE to identify all published articles on the economic implications of ADHD, and authors were contacted to locate conference abstracts and articles in press that were not yet indexed. In total, 22 relevant items were located including published original studies, economic review articles, conference presentations, and reports available on the Internet. All costs were updated and presented in terms of year 2004 US dollars. A growing body of literature, primarily published in the United States, has demonstrated that ADHD places a substantial economic burden on patients, families, and third-party payers. Results of the medical cost studies consistently indicated that children with ADHD had higher annual medical costs than either matched controls (difference ranged from $503 to $1,343) or non-matched controls (difference ranged from $207 to $1,560) without ADHD. Two studies of adult samples found similar results, with significantly higher annual medical costs among adults with ADHD (ranging from $4,929 to $5,651) than among matched controls (ranging from $1,473 to $2,771). A limited number of studies have examined other economic implications of ADHD including costs to families; costs of criminality among individuals with ADHD; costs related to common psychiatric and medical comorbidities of ADHD; indirect costs associated with work loss among adults with ADHD; and costs of accidents among individuals with ADHD. Treatment cost-effectiveness studies have primarily focused on methylphenidate, which is a cost-effective treatment option with cost-effectiveness ratios ranging from $15,509 to $27,766 per quality-adjusted life year (QALY) gained. As new treatments are introduced it will be important to evaluate their cost-effectiveness to provide an indication of their potential value to clinicians, patients, families, and third-party payers.

## Introduction

Attention-deficit hyperactivity disorder (ADHD) is characterized by a persistent and developmentally inappropriate pattern of inattention, hyperactivity, and/or impulsivity [1]. Children with ADHD tend to have difficulty organizing tasks and sustaining attention during schoolwork or play activities. Typical disruptive behaviors include failing to remain seated, talking excessively, playing noisily, and blurting out answers before questions have been completed. ADHD is relatively common, with prevalence rates among school-age children in the United States ranging from roughly 4% to 12%, depending on the diagnostic approach [2-4]. Furthermore, the percentage of children treated for ADHD in the United States increased dramatically from the 1980s to the 1990s [5,6].

ADHD is associated with impairment in many areas of children's lives, including academic performance, social functioning, and overall quality of life [7-12]. Children with ADHD are frequently rejected by their peers as early as the first day of contact, likely as a result of their tendency toward disruptive and aggressive behavior [13]. ADHD also has long-term negative outcomes for many children, including decreased educational attainment, work performance, and occupational stability compared to individuals without ADHD [14,15].

Because of the broad impact of ADHD, the disorder is likely to have serious economic implications for children, families, and society. Research has only recently begun to examine these economic costs, but the initial studies suggest that ADHD leads to increased costs in healthcare and other domains. The purpose of this paper is to review and summarize available literature on the economic costs of ADHD, as well as potential economic benefits of treating this condition. In addition, recommendations for additional research on the economic implications of ADHD are provided.

## Methods

A literature search was performed using MEDLINE, accessed through PubMed, to identify all published articles on the economic implications of ADHD. Searches were conducted using both the term and medical subject heading "ADHD" (in addition to the full term "attention-deficit/hyperactivity disorder" as well as individual words including "attention" and "hyperactivity"), along with economic terms including "cost," "costs," "economic," and "economics." Reference sections of relevant articles, including one review article that summarized eight economic studies [16], were reviewed to identify additional studies that may not have been included in MEDLINE. Finally, authors were contacted to locate conference abstracts and articles in press that were not yet indexed. In total, 22 relevant items were located including published original studies, unpublished conference presentations, and reports available on the Internet. Articles that discussed resource use patterns for ADHD but did not attach costs to these patterns [17] are not included in this review. All costs were updated to year 2004 US dollars based on the medical component of the Consumer Price Index [18].

## Results

### Direct Medical Costs of ADHD in Children and Adolescents

Formal cost of illness studies measure the "economic" burden resulting from disease and illness across a defined population (e.g., patients in the US), including both direct medical costs and indirect (e.g., lost productivity) costs [19]. Although no formal cost of illness estimates incorporating both direct and indirect costs of ADHD have been published, several studies have presented estimates of the direct medical costs of treating children and adolescents with ADHD (see Table 1). In three of the studies [20-22], annual costs of care for children with ADHD were compared to annual costs for matched (on age and sex) controls. Three other studies included a similar comparison, but utilized non-matched controls [23-25]. Studies by Chan et al. [26] and Kelleher et al. [27] compared treatment costs for childhood ADHD with childhood asthma. Leslie et al. [28] examined trends over time in costs for children with ADHD, and Marchetti et al. [29] compared costs by type of ADHD treatment. Birnbaum et al. [30] used the data set previously analyzed by Swensen et al. [21,22] to estimate total excess costs for the US population, defined as the difference between ADHD patients and matched controls.

**Table 1:** Studies of the Direct Costs of ADHD

**Citation**	**Sample**	**Data Sources (Time Period)**	**Findings^1^**
Birnbaum et al. 2005	Treated ADHD patients aged 7–44 (N = 1219) and their family members under age 65 (N = 3692); controls without ADHD matched to both patients (N = 1219) and family members (N = 3692) on age, gender, employment status, geographical location age, gender, state of residence, and employment status)	Claims data from large Fortune 100 company (1996–1998)	This study estimated total excess costs for the US population, defined as the difference between ADHD patients/family members and controls. Annual mean direct ADHD treatment costs (2004 US $) were $674/$745 for girls/boys with ADHD (excess costs = $0.53/$1.06 billion) and $412/$529 for female/male adults with ADHD (excess costs = $0.13/$0.40 billion). Annual other direct treatment costs (2004 US $) were $865/$990 for girls/boys (excess costs = $0.80/$2.0 billion) and $2609/$3022 for female/male adults with ADHD (excess costs = $0.67/$1.46 billion).
Burd et al. 2003a	Children aged 0–21 years with ADHD (N = 3,872) and non-matched controls (N = 95,119) without ADHD	North Dakota Health Claims Database (1996–1997)	Annual, mean direct treatment costs (2004 US $) were $870 for ADHD patients versus $663 for controls; 1.9% of total annual health expenditures in North Dakota attributable to ADHD.
Chan et al. 2002	Children aged 5–20 years with ADHD (N = 165), asthma (N = 322) or neither diagnosis (N = 4,952)	Nationally representative household survey data (1996)	Annual, incremental direct treatment costs (2004 US $) were $661 for children with ADHD (*P *< 0.001) and $603 for children with asthma (*P *< 0.01) (relative to costs for children with neither diagnosis)
Guevara et al. 2001	Children aged 3–17 years with ADHD (N = 2992) and matched (on age and sex) controls (N = 11,968) without ADHD	Health maintenance organization in western Washington State (1997)	Annual, incremental direct treatment costs (2004 US $) were $503 (95% CI: $450–552) for children with ADHD alone and $1088 (95% CI: $899–$1,304) for children with ADHD plus coexisting mental health disorders (relative to costs for children with no ADHD)
Kelleher et al. 2001	Children aged 7–20 years with ADHD (N = 1,602) and with asthma (N = 1,411)	Medicaid claims data for patients in Pittsburgh, PA and surrounding counties (1994–1995)	Annual, mean (± SD) direct treatment costs (2004 US $) were $2,567 ± $2,959 for the ADHD group versus $2,382 ± $2,664 for the asthma group (difference not statistically significant)
Leibson et al. 2001	Children aged 5–19 years with ADHD (N = 309) and non-matched controls (N = 3,810) without ADHD	Rochester, Minnesota medical facility-linked billing data system (1987–1995)	Long-term (9 year), median direct treatment costs (2004 US $) for ADHD patients compared with those without ADHD were more than double ($6,158 vs. $2,780; *P *< 0.001), even for the subset with no hospital or emergency room admissions ($183 vs. $93; P < 0.001)
Leslie et al. 2001	Children aged ≤ 17 years with use of mental health services, including patients with hyperactivity (N~10,000)	Health care claims for privately insured population (MarketScan^®^) (1993–1996)	Annual inpatient costs (2004 US $) per treated hyperactive patient declined from $26,550 in 1993 to $8,644 in 1996 (*P *< 0.001); there was also a significant decline in outpatient treatment costs ($931 and $659, respectively, *P *< 0.001)
Mandell et al. 2003	Children aged 3–15 years with ADHD (N = 4,306) and with no psychiatric disorder (N = 60,975)	Medicaid claims data for patients in Philadelphia, PA (1993–1996)	Long-term (3 year), mean direct treatment costs (2004 US $) were $4,891 for ADHD patients versus $221 for patients with no psychiatric disorder
Marchetti et al. 2001	Hypothetical cohort of school-aged children with ADHD	Literature review, managed care survey, clinical experts (2000–2001)	Average, total annual expected cost (2001 US $) per treated patient was $1,710 for Metadate CD, $1876 for Concerta, $2,061 for methylphenidate immediate-release/extended release (MPH IR/ER), $2,122 for MPH IR, $2,392 for Ritalin, and $2,567 for Adderall.
Secnik et al. 2005b	Adults aged 18–64 with ADHD (N = 2,252) and matched controls without ADHD (N = 2252; matched on age, gender, metropolitan statistical area, and type of insurance coverage)	Health care claims for privately insured population (MarketScan^®^) (1999–2001)	Controlling for the impact of comorbidities, adults diagnosed with ADHD had significantly (*P *< 0.0001) higher outpatient costs ($3,009 vs. $1.491), inpatient costs ($1,259 vs. $514), prescription drug costs ($1,673 vs. $1,008) and total annual medical costs ($5,651 vs. $2,771) compared with matched controls without ADHD
Swensen et al. 2003	Children aged 0–18 years with ADHD (N = 1,086) and matched (on age, gender, and state of residence) controls (N = 1,086) without ADHD	Claims data from large Fortune 100 company (1996–1998)	Annual, mean (± SD) direct treatment costs (2004 US $) were $2,046 ± $3,474 for the ADHD group versus $703 ± $2,215 for matched controls without ADHD (*P *< 0.0001).
Swensen et al. 2004	Individuals aged 0–64 with ADHD (N = 1,308) and matched (on age, gender, state of residence, and employment status) controls (N = 1,308) without ADHD	Claims data from large Fortune 100 company (1996–1998)	Annual, mean direct treatment costs (2004 US $) were $1,797 for children with ADHD versus $577 for matched controls without ADHD (*P *< 0.05); $2,230 for adolescents with ADHD versus $783 for matched controls without ADHD (*P *< 0.05); and $4,929 for adults with ADHD versus $1,473 for matched controls without ADHD (*P *< 0.05).

^1^All costs updated to Year 2004 US $ using the Medical Services component of the Consumer Price Index (for US-based studies). For non-US studies, all country-specific costs first updated to Year 2004 currency values based on country-specific inflators; and then converted to Year 2004 US$ based on currency exchange rates.

MPH = methylphenidate

IR = immediate release

ER = extended release

A majority of the medical cost studies used insurance claims data from private insurers [20-22,28,30], from state Medicaid agencies [25,27], or from both sources [23,24]. The cost estimates reported by Chan et al. [26] were based on nationally representative household survey data, while the study by Marchetti et al. [29] relied on literature review and clinical expert opinion. The time period for the health care resource data used in the various studies ranged from 1987 to 1998.

The results of the medical cost studies were consistent in indicating that children with ADHD had higher annual medical costs than either matched controls (difference ranged from $503 to $1,343) or non-matched controls (difference ranged from $207 to $1,560) without ADHD (Table 1). The higher costs for ADHD patients were due to increased use of hospitalizations, primary care office visits, outpatient mental health visits, and pharmacy fills. For example, children with ADHD were 9.02 and 8.75 times more likely than other children (matched on age and sex) to have outpatient mental health visits and pharmacy fills, respectively [20]. When compared with cohorts of children with asthma (who were more likely to be female and African-American), children with ADHD had slightly higher annual treatment costs; but the differences were not statistically significant [26,27]. When Birnbaum and colleagues [30] used claims data to estimate excess costs of ADHD across the US population, the excess ADHD-related treatment costs were $0.53 billion for girls and $1.06 billion for boys, and the excess overall healthcare costs were $0.80 billion for girls and $2.00 billion for boys.

Marchetti et al. [29] examined the costs of six different ADHD drug therapies: generic and branded (Ritalin) MPH immediate release (IR) therapies, two branded MPH extended release (ER) therapies (Concerta and Metadate CD), generic MPH IR/ER, and a combination therapy of amphetamine salts (Adderall). The annual expected cost of treatment with each drug therapy, including costs incurred with physician visits and lab exams, was highest for Adderall at $2,567 (2004 US $) and lowest for Metadate CD at $1,710. The cost for the other therapies ranged from $2,061 (Concerta) to $2,392 (Ritalin).

### Direct Medical Costs of ADHD in Adults

Although ADHD is often perceived as a disorder of childhood and adolescence, there is growing awareness that many children continue to demonstrate symptoms in adulthood, although many adults with ADHD remain undiagnosed and untreated [31]. Limited prevalence data on adult ADHD are available, but estimates generally indicate that 30% to 70% of children with ADHD continue to have symptoms in adulthood [32,33]. Adult symptom presentation is likely to be somewhat different from the common symptoms of childhood, with less hyperactivity, but with continued problems with organizational tasks and distractibility. ADHD is known to have a broad range of negative outcomes for adults, including relatively high rates of criminality, poor job performance, lower occupational status, problems in social skills, poor driving records, and comorbid psychiatric disorders [14,15,34].

Despite the continuing impact of ADHD in adulthood, only three studies were located that examined economic costs of adults with ADHD (Table 1) [22,30,35]. Secnik et al. and Swensen et al. compared costs of care for adults with ADHD with matched controls (matched on age, sex, metropolitan statistical area, and type of insurance coverage), and both utilized privately insured claims data for the analysis. The two studies both indicated that adults with ADHD have significantly higher annual medical costs (ranging from $4,929 to $5,651) than matched controls (ranging from $1,473 to $2,771), even after controlling for patient comorbidity [35]. Birnbaum and colleagues used the same sample as Swensen et al. to estimate total excess costs for adults in the US population (i.e., the difference between ADHD patients and matched controls). The excess ADHD-related treatment costs were $0.13 billion for women and $0.40 billion for men, and the excess overall healthcare costs were $4.79 billion for women and $8.51 billion for men.

### Costs to Families

The relationship between children and their families is complex and bi-directional, involving simultaneous mutual influence [36,37]. Children are shaped by their experiences with parents, while simultaneously influencing their parents' behavior and emotions [38,39]. This mutual influence between parents and children has been well-documented among families with a child who has been diagnosed with ADHD. Family environment and parent-child interaction has been shown to be a key causal factor in the development of ADHD and related conduct problems in longitudinal studies [40]. Conversely, the symptoms of ADHD have profound effects not only on the child, but also on the child's parents. For example, children's ADHD is frequently linked with strain in the parent-child relationship, disturbance in parents' marital functioning (e.g., less marital satisfaction and more conflict than parents of children without ADHD), and extremely high parental stress [40,41].

A recent study conducted by Swensen and colleagues [21] suggests that childhood ADHD also places an economic burden on parents and other family members. This analysis was conducted using 1996–1998 data from a national sample of over 100,000 beneficiaries of a large US company that include industrial, service, and professional employees. Family members of individuals affected with ADHD had 1.6 times as many medical claims as matched control individuals without a family member diagnosed with ADHD (matching based on age, gender, geographical location, and employment status). This greater healthcare utilization resulted in increased costs. Annual direct per-capita medical costs were twice as much for family members of ADHD patients ($2,740) than for family members of control patients ($1,365). Indirect costs related to disability and absenteeism followed a similar pattern (family members of ADHD patients, $888; family members of controls, $551). Birnbaum et al. [30] used the same data set to estimate excess healthcare costs across the US population (i.e., the difference between family members of ADHD patients and family members of matched controls), which were $6.78 billion for family members of children with ADHD and $12.10 billion for family members of adults with ADHD.

There are several possible reasons for the higher indirect costs of parents whose children have ADHD. For example, children with ADHD are likely to require energy and attention that might otherwise be focused on work-related responsibilities. Furthermore, these parents may often be required to miss work in order to meet with teachers or take their children to appointments with physicians or mental health professionals. These high indirect costs suggest that ADHD has a financial impact not only on family members, but also on employers who might be affected by family members increased disability and absenteeism.

A nationally representative US survey conducted in 1998 suggests that mothers of children with ADHD perceive the financial impact of their children's disorder [42]. Compared with mothers whose children were not diagnosed with ADHD, mothers of children with ADHD were 3.3 times as likely to say that the family could not afford prescription medication for the child and 7.4 times as likely to say the family could not afford mental-health care for the child. Another study of parent perceptions clearly demonstrates the substantial economic burden of ADHD [43]. In this study, parents were asked whether they perceive their child's hyperactivity as a serious problem. The strongest predictor of whether parents considered ADHD to be a serious problem was the financial impact related to work, defined as the impact of the child's behavior on either parent's employment patterns or chances of a career (e.g., leaving work to pick up the child). Compared with parents who did not think ADHD was a serious problem, parents who perceived their child's ADHD to be a serious problem were 17.6 times more likely to say that there child's ADHD had a financial impact related to their work. Taken together, Swensen's study of family medical costs and these two studies of parent perceptions indicate that ADHD has a substantial impact on family finances.

Given the impact of a child's ADHD on parents' absenteeism and productivity, it may be beneficial for employers and human resource specialists to consider strategies for minimizing this impact. A recent survey of 41 employers in four American cities found that the participants knew little about ADHD prevalence or its potential effects on parents, despite their responsibility for purchasing employees' health insurance [44]. However, employers did offer several benefits and policies that could be helpful to parents whose children have ADHD, including on-site parent training programs, assistance with child care, flexible work/leave policies, and referral services that linked parents with community programs. When asked about benefits that could be targeted specifically for families of children with ADHD, employers suggested lunch seminars about ADHD and flexible hours for employees to meet with schools or physicians. As employers gain greater awareness of the economic impact of ADHD, it is hoped that such programs and benefits may be implemented at more companies.

### Costs of Criminality

Several longitudinal studies have shown that childhood ADHD is associated with criminality in adolescence and adulthood. For example, a study conducted in Los Angeles found that children diagnosed with ADHD between the ages of 6 and 12 years old had significantly higher juvenile (46% versus 11%) and adult (21% versus 1%) arrest rates compared to normal control subjects [45]. A similar study conducted in New York found that children with ADHD were more likely than controls to later be arrested (39% versus 20%), convicted (28% versus 11%), and incarcerated (9% versus 1%) [46]. Another study, conducted with 17–18 year old adolescents in San Francisco, found that the ADHD group was more likely than the control group to be on probation, in jail, or assigned to a social worker by the court [47].

One study has estimated the economic impact of criminality associated with ADHD [48]. Data were from a sample of children (4–12 years old) identified in 1979 and 1980. Follow-up interviews were conducted between 1991 and 1996 with 149 children diagnosed with ADHD and 76 control children when a sample ranged in age from 19 to 25 years. Criminal history was assessed through self-report, including crimes (e.g., stealing, assault), juvenile detention, probation, and jail. The costs of crimes incurred by victims and costs to the criminal justice system were estimated based on information from the Bureau of Justice Statistics, the Federal Bureau of Investigation, and the Criminal Justice Institute. Compared with the control group, the ADHD patients were more than twice as likely to have been arrested (48% versus 20%). The mean total criminal costs were dramatically greater for ADHD patients than for controls ($12,868 versus $498). All differences were statistically significant. Although this study should be considered a rough estimate, findings strongly suggest that criminality associated with ADHD results in a significant cost to society.

### Costs of Comorbidities

Children with ADHD tend to have elevated rates of other psychiatric conditions [21,49,50] For example, about 30% of children with ADHD meet criteria for an anxiety disorder, compared to about 10% of the general population. Behavioral problems such as conduct disorder and oppositional defiant disorder are particularly common among children with ADHD, with comorbid rates of roughly 50%. Other conditions that are commonly comorbid with ADHD include learning disabilities, depression, and possibly bipolar disorder. When estimating the impact of ADHD, it is important to consider these comorbidities because comorbid conditions can influence children's long-term course and response to treatment.

One study has estimated the incremental increase in costs of treatment for ADHD with comorbid conditions, compared with treatment of ADHD alone [51]. Analyses were conducted using 1996 and 1997 data from the North Dakota Department of Health's Claims Database. Generally, comorbid psychiatric disorders substantially increased the costs of treating children with ADHD. For example over the two years, comorbid depression increased costs by an average of $358 per patient per year. Increases were also observed with oppositional defiant disorder ($258), bipolar disorder ($541), conduct disorder ($488), anxiety ($499), nondependent drug use ($868), tics ($198), and personality disorders ($247). Non-psychiatric medical disorders also resulted in increased costs, including respiratory illness ($630), acute sinusitis ($670), general injuries ($972), and allergies ($507).

In the North Dakota sample, the only comorbid conditions that were associated with lower costs compared to children without comorbid conditions were learning disabilities (-$759) and epilepsy (-$777). However, another recent study focusing on comorbid epilepsy in an administrative claims database from 1998 to 2001 found contrasting results [52]. In a sample, the average annual treatment costs for children with ADHD and comorbid epilepsy were $5,194, compared with $4,246 for children with ADHD but not epilepsy. Despite the mixed results relating to learning disabilities and epilepsy, initial findings suggest that the common comorbid conditions of ADHD may contribute to elevated treatment costs among this population.

### Costs of Accidents

Children with ADHD have been shown to be more accident prone than other children [53], likely because of their tendencies toward impulsive, overactive behavior. They are also more likely than other children to experience injuries due to accidents, such as broken bones, lacerations, head injuries, bruises, lost teeth, or accidental poisonings [54,55]. One study has estimated the incidence and cost of accidents among individuals with ADHD using an administrative database of medical, pharmaceutical, and disability claims for national manufacturers' employees, spouses, dependents, and retirees [22]. Analyses were conducted for the whole population, adults alone, children under age 12, and adolescents aged 12 to 18 years. ADHD patients in all age groups were more likely than a matched control group to have at least one accident claim: children, 28% versus 18%; adolescents, 32% versus 23%, and adults, 38% versus 18%. Among adults, the accident-specific direct medical costs were significantly higher among ADHD patients than among the control group ($642 versus $194). Among children and adolescents, there were not significant differences in accident-specific costs between the ADHD groups and the control groups.

### Costs of Work Loss

ADHD is associated with work-related problems in adulthood such as poor job performance, lower occupational status, less job stability, and increased absence days in comparison to adults without ADHD [15,34,35,56]. This poor performance and work loss is likely to have profound economic implications. One study quantified this impact by estimating the excess costs (i.e., the difference between adult ADHD patients and matched controls) related to work loss [30]. Indirect work loss costs were calculated based on employer payments for disability claims and imputed wages for medically-related work absence days (e.g., days in the hospital, physician visits). The excess costs were $1.20 billion for women with ADHD and $2.26 billion for men with ADHD.

### Cost-Effectiveness of Treatments for ADHD

Three published studies have utilized decision analytic modeling techniques to assess the cost-effectiveness of drug therapy (i.e. methylphenidate [MPH]) for ADHD [57-59] (see Table 2). Each of these studies represented "complete" economic evaluations (i.e. estimated both the incremental costs and incremental effects associated with treatment). In two of the studies [57,58], the effectiveness of treatment was measured in terms of "quality-adjusted life years" (QALYs), an outcome measure that incorporates quality of life benefits and time [60]. These quality of life benefits are quantified using utility scores, which have been shown to be feasible and valid for assessment of children with ADHD [61-63]. In the third study [59], treatment effectiveness was based on gains in the Conners Teacher Rating Scale, a commonly used teacher-report questionnaire for assessing children's classroom behavior [64].

**Table 2:** Studies of the Cost-Effectiveness of Treatment for ADHD

**Citation**	**Comparators**	**Study Design**	**Findings^1^**
Gilmore & Milne 2001	MPH, placebo	• Decision analytic model assessing cost-utility	Cost per each additional QALY gained with MPH treatment (versus no treatment) ranged from $15,509 to $19,281 when considering short-and medium-term benefits of MPH. Cost per QALY gained ranged from $9,850–$59,101 in sensitivity analyses
Novartis data on file (2000; referenced in Lord & Paisley 2000)	MPH, placebo	• Decision analytic model assessing cost-utility	Cost per each additional QALY gained with MPH treatment (versus no treatment) was $27,766.
Zupancic et al. 1998	MPH, placebo	• Decision analytic model assessing cost-effectiveness	Cost per each additional point in the Conners Teacher Rating Scale gained with MPH treatment (versus no treatment) was $93, or $560 for a 6-point (1 SD) gain.

^1^All costs updated to Year 2004 US $ using the Medical Services component of the Consumer Price Index (for US-based studies). For non-US studies, all country-specific costs first updated to Year 2004 currency values based on country-specific inflators; and then converted to Year 2004 US$ based on currency exchange rates.

MPH = methylphenidate

QALY = quality-adjusted life years

Overall, results of the three modeling analyses indicate that MPH is a cost-effective treatment option for children with ADHD. The cost per QALY gained in the Gilmore and Milne [57] study ranged from $15,509 to $19,281 when considering the short-and medium-term benefits of MPH. The authors note that evidence of cost-effectiveness beyond 6 months is poorer, and it is uncertain whether the effects of MPH persist into adolescence and adulthood. In the Novartis study, the cost per QALY gained was $27,766.

## CONCLUSIONS AND RECOMMENDATIONS FOR FUTURE RESEARCH

A growing body of literature has demonstrated that ADHD places a substantial economic burden on patients, families, and third-party payers. However, all available published studies on the economic implications of ADHD are relatively recent, and there are many additional questions to be examined in future research. Thus far, studies have identified several aspects of ADHD that are likely to have economic implications, including direct treatment costs, increased rates of comorbid psychiatric disorders, high accident rates, work loss, and criminality. There are many other well-documented outcomes of ADHD that are likely to have economic implications. For example, ADHD frequently has detrimental effects on a child's academic performance and behavior in school. These difficulties are likely to place an economic burden on school systems, as there is an increased need for school-based services such as in-school medication administration; special education services; child and possibly parent counseling; educational testing; development of individualized educational programs; and efforts to address disruptive classroom behaviors [65,66]. Research is needed to quantify these costs and identify strategies for implementing the most cost-effective services. Furthermore, it is well known that adults with ADHD tend to have poor driving records and relatively high rates of traffic accidents [14,15,34]. These driving problems are also likely to present a significant cost which is not yet been examined.

The international literature on cost of ADHD could also be expanded. Nearly all published studies identified for the current review were conducted in the US. Given international differences in medical care systems and practice patterns, it is difficult to apply the direct treatment costs from US studies to countries outside the US. It is likely, however, that the indirect cost burden of ADHD identified in US studies may be similar to the burden in other countries, although it may not be recognized to the same extent. Recognition, diagnosis, and treatment of ADHD are increasing in Europe and Australia, and future studies may document the economic burden of ADHD in these areas.

Finally, more work is needed on determining the potential cost-effectiveness of the various treatment options for ADHD. The initial research on cost-effectiveness of treatment has focused on MPH, and results generally indicate that treatment of ADHD is cost-effective. As new treatments are introduced (e.g., the new non-stimulant atomoxetine), it is important to evaluate their cost-effectiveness to provide an indication of their potential value to clinicians, patients, families, and third-party payers. Effective treatments, while possibly increasing direct medical costs, are likely to reduce the overall burden of ADHD by controlling symptoms, improving children's functioning, and substantially reducing indirect costs to families.

## List of Abbreviations

Attention-deficit hyperactivity disorder (ADHD)

extended release (ER)

immediate release (IR)

methylphenidate (MPH)

quality-adjusted life year (QALY)

## Competing interests

All three authors of this article are employed at the MEDTAP Institute at UBC, which is an independent health services research organization. Funding for the current review was provided by Eli Lilly & Co.

## Authors' contributions

LM was the principal investigator. He conducted the literature search, designed the structure of the manuscript, reviewed and integrated much of the literature, and wrote and conceived most of the manuscript. CP and MP had primary responsibility for the two sections that review, integrate, and summarize studies of direct costs and treatment cost-effectiveness. They reviewed the relevant literature, wrote these two important sections of the paper, and created Tables 1 and 2.
